# Neuroprotective Epigenetic Changes Induced by Maternal Treatment with an Inhibitor of Soluble Epoxide Hydrolase Prevents Early Alzheimer′s Disease Neurodegeneration

**DOI:** 10.3390/ijms232315151

**Published:** 2022-12-02

**Authors:** Clara Bartra, Alba Irisarri, Ainhoa Villoslada, Rubén Corpas, Samuel Aguirre, Elisa García-Lara, Cristina Suñol, Mercè Pallàs, Christian Griñán-Ferré, Coral Sanfeliu

**Affiliations:** 1 Institut d′Investigacions Biomèdiques de Barcelona (IIBB), Consejo Superior de Científicas (CSIC), 08036 Barcelona, Spain; 2 Institut d′Investigacions Biomèdiques August Pi i Sunyer (IDIBAPS), 08036 Barcelona, Spain; 3 Department of Pharmacology and Therapeutic Chemistry, Faculty of Pharmacy and Food Sciences, 08028 Barcelona, Spain; 4 Institut de Neurociències, Universitat de Barcelona, 08028 Barcelona, Spain

**Keywords:** Alzheimer′s disease, prenatal risk, soluble epoxide hydrolase, maternal TPPU, epigenetics, long-term neuroprotection, memory, neuroinflammation, 5XFAD mice

## Abstract

Modulation of Alzheimer′s disease (AD) risk begins early in life. During embryo development and postnatal maturation, the brain receives maternal physiological influences and establishes epigenetic patterns that build its level of resilience to late-life diseases. The soluble epoxide hydrolase inhibitor N-[1-(1-oxopropyl)-4-piperidinyl]-N′-[4-(trifluoromethoxy)phenyl] urea (TPPU), reported as ant-inflammatory and neuroprotective against AD pathology in the adult 5XFAD mouse model of AD, was administered to wild-type (WT) female mice mated to heterozygous 5XFAD males during gestation and lactation. Two-month-old 5XFAD male and female offspring of vehicle-treated dams showed memory loss as expected. Remarkably, maternal treatment with TPPU fully prevented memory loss in 5XFAD. TPPU-induced brain epigenetic changes in both WT and 5XFAD mice, modulating global DNA methylation (5-mC) and hydroxymethylation (5-hmC) and reducing the gene expression of some histone deacetylase enzymes *(Hdac1* and *Hdac2*), might be on the basis of the long-term neuroprotection against cognitive impairment and neurodegeneration. In the neuropathological analysis, both WT and 5XFAD offspring of TPPU-treated dams showed lower levels of AD biomarkers of tau hyperphosphorylation and microglia activation (*Trem2*) than the offspring of vehicle-treated dams. Regarding sex differences, males and females were similarly protected by maternal TPPU, but females showed higher levels of AD risk markers of gliosis and neurodegeneration. Taken together, our results reveal that maternal treatment with TPPU impacts in preventing or delaying memory loss and AD pathology by inducing long-term modifications in the epigenetic machinery and its marks.

## 1. Introduction

Therapies for Alzheimer′s disease (AD) have been elusive despite some recent promising clinical trials with monoclonal antibodies against amyloid beta. However, all disease-modifying therapies assayed so far showed minor or no neuroprotective effects [1]. Many new drugs are in the pipeline, but prevention should be urgently addressed [2]. Early environmental influences are increasingly considered as modulators of late-life neurodegeneration and AD risk [3]. During gestation and breastfeeding, several maternal factors such as diet, consumption of drugs, and stress can affect the baby’s brain development. An impact on plasticity and epigenetically-mediated programming of diverse brain cells has been proposed linking early influences and the late risk of neurodegenerative disorders [4,5]. Epigenetic mechanisms regulate gene transcription through DNA methylation and other DNA modifications, a variety of post-translational modifications of histones, chromatin remodelers, and several classes of non-coding RNAs. The imprinting of the genome by epigenetic marks is a dynamic process that can be modified by environmental factors such as drugs, nutrition, and mental stimulation [6]. Dysregulation of the brain epigenome has been strongly associated with memory impairment and neurodegeneration [7]. Accordingly, epigenetic mechanisms play a critical role in the genome–environment interaction in AD [8]. Likewise, maternal exposure to different environmental factors can affect the offspring cognitive outcome for the next generation [9]. For instance, experimental studies with a maternal diet supplemented with choline or with resveratrol have shown the relevance of the triggered epigenetic mechanisms in the long-term neuroprotection of the offspring of mouse models of cognitive impairment [10,11]. Specifically, choline is a major dietary donor of methyl groups essential for DNA and histone methylation that play an important role in fetal brain development [12]. Epigenetic mechanisms of maternal choline induce long-term changes in gene expression levels affecting neurotransmission, neurotrophism, and autophagy pathways in the Ts65Dn mouse model of Down′s syndrome [10]. Moreover, resveratrol is a natural polyphenol with potential therapeutic use in neurodegenerative diseases that modulates DNA methyl transferase and histone deacetylase enzymes among other epigenetic mechanisms [13]. Similarly, maternal treatment with resveratrol induces epigenetic regulation of redox master genes, autophagy, and mitochondrial biogenesis leading to cognitive improvement in the offspring of the senescent mice SAMP8 [11]. Thus, research on treatments affecting neurodevelopmental epigenetics may lead to new therapeutic strategies against AD. This may be particularly relevant when known risk factors are present, such as in families bearing risk alleles or mild late-onset AD mutations [14].

In a search for epigenetic-mediated therapies, we hypothesized that an anti-inflammatory drug that is neuroprotective in adult mouse models of AD would induce beneficial epigenetic imprinting of the innate immune system during embryonic and perinatal brain development. As a novel anti-inflammatory drug, we and others have shown that 1-(1-propanoylpiperidin-4-yl)-3-[4-(trifluoromethoxy)phenyl]urea (TPPU), an inhibitor of the soluble epoxide hydrolase (sEH) enzyme that converts anti-inflammatory oxylipins epoxyeicosatrienoic acids (EETs) to the corresponding less active dihydroxyeicosatrienoic acids (DHETs), is neuroprotective in experimental models of cognitive loss and dementia [15,16,17,18,19,20]. Furthermore, EETs have been associated with epigenetic changes in differentiating adipocyte cells leading to decreased inflamed adipocyte phenotypes [21]. Additionally, in terms of maternal diet, EETs are produced from the n-6 polyunsaturated fatty acid (PUFA) arachidonic acid and have important roles in the regulation of inflammation and immune response, vascular enhancement, and specific neuroprotective effects [22,23,24,25]. In general, PUFAs are essential fats for brain development and function. PUFAs are found in plant and animal foods, such as salmon, vegetable oils, and some nuts and seeds. PUFAs are metabolized to several families of oxylipins that are the main mediators of the beneficial effects [26]. In adults, alterations in fatty acid metabolism have been reported in AD patients and mouse models of AD [27]. Furthermore, lipidomic alterations have been associated with epigenetic changes and AD development [28].

In this study, pharmacological inhibition of sEH was induced in female wild-type (WT) mice mated with male heterozygous 5XFAD mice by TPPU treatment. Six WT females were dosed orally with 5 mg/kg/day of TPPU added to their drinking water and six WT females were dosed with the cyclodextrin vehicle. Treatment started at mating and was maintained during pregnancy and lactation. The litter pups that were raised together were either WT or transgenic AD mice of the 5XFAD strain. Male and female mice were included in the study. Our aim was to investigate the long-term protective effects of maternal TPPU dosing in young adult 5XFAD mice compared to WT mice, emphasizing epigenetic changes that may impact the early course of AD. To this end, we fully analyzed the offspring at two months of age, when 5XFAD mice show the first pathological features of AD. As a reference, we included a group of untreated 7-month-old male 5XFAD mice with advanced AD pathology for analysis of selected markers.

## 2. Results

### 2.1. Maternal TPPU Treatment Prevented Cognitive and Behavioral Changes of 5XFAD Offspring

#### 2.1.1. Protection against Memory Loss

**Figure 1:**
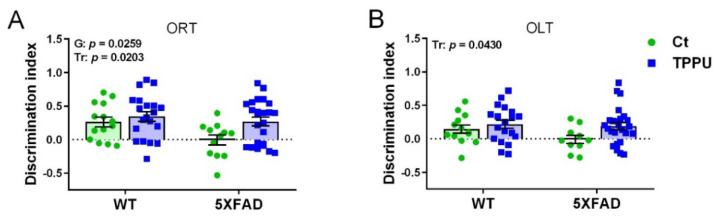
Cognitive testing in 2-month-old WT and 5XFAD mice from litters with maternal TPPU treatment or control (Ct). Breeding WT female mice were mated to 5XFAD male mice and dosed with 5 mg/kg/day of TPPU or vehicle during pregnancy and lactation. (**A**) Object recognition test (ORT); (**B**) object location test. Experimental maternal treatment: Ct (green circles), TPPU (blue squares); mouse genotype: WT (wild-type mice), 5XFAD (AD 5XFAD transgenic strain). Values are mean ± SEM of male and female mice ((**A**) *n* = 12−20; (**B**) *n* = 10−25). Statistics: two-way ANOVA. There was a significant effect of the treatment factor (Tr) in (**A**,**B**) and of genotype factor (G) in (**B**). *p* values of the significant factors are indicated in the corresponding graphs.

Two-month-old male and female 5XFAD mice from control litters whose dams were treated with vehicle (Ct) showed memory impairment in the object recognition test (ORT) and the object location test (OLT), as shown in Figure 1A,B. Interestingly, 5XFAD mice belonging to litters with maternal TPPU treatment (TPPU) showed absence of impairment, although the genotype factor did not reach significance in OLT. Furthermore, TPPU induced cognitive enhancement in WT mice, as shown by the statistical significance of the treatment factor in the two-way ANOVA. Thus, maternal TPPU protected against learning and memory impairment in two different paradigms: the recognition of a new object versus a familiar one and the recognition of a change in object position.

#### 2.1.2. Maternal Treatment with TPPU Rescued Exploratory Activity in the 5XFAD Offspring

**Figure 2:**
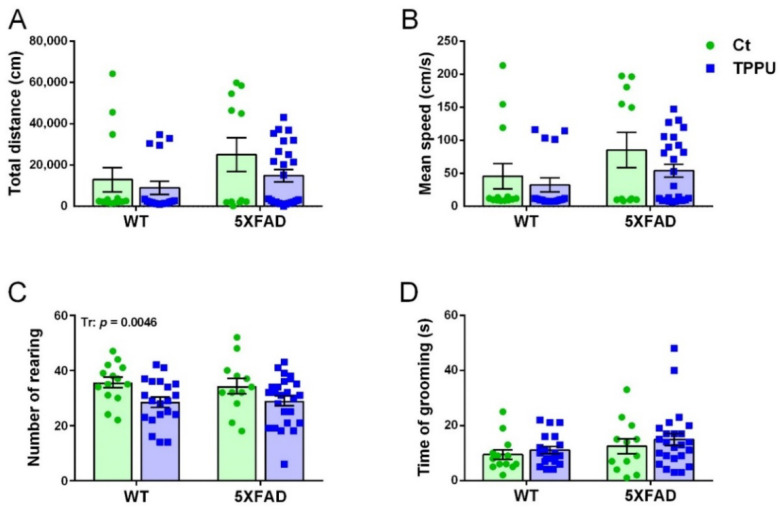
Exploratory behavior in the open field test of 2-month-old WT and 5XFAD mice from litters with maternal TPPU treatment or control (Ct). (**A**) Horizontal activity; (**B**) mean speed without rest intervals; (**C**) vertical activity; (**D**) grooming activity. Values are mean ± SEM of male and female mice ((**A**–**C**) *n* = 12–25; (**D**) *n* = 12–24). Statistics: ANOVA, significant effect of treatment (Tr) in (**C**), *p* value of the significant factor is indicated in the corresponding graph; borderline significant effect of genotype (G) in (**A**,**B**) (see text).

**Figure 3:**
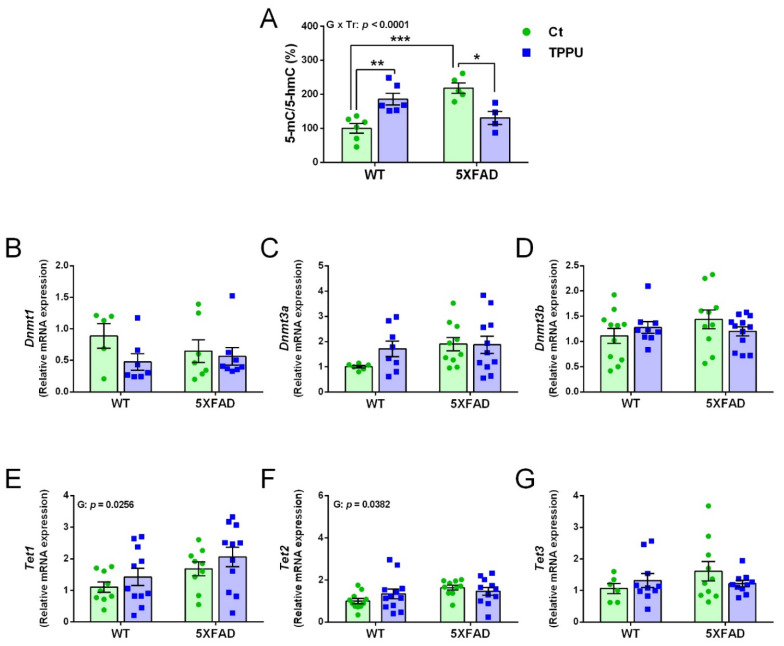
DNA methylation and demethylation changes in 2-month-old WT and 5XFAD mice from litters with maternal TPPU treatment or control (Ct). (**A**) Methylation profile obtained by the 5-methylcytosine (5-mC) and 5-hydroxymethylcytosine (5-hmC) ratio (%); (**B**–**D**) expression levels of the DNA methyltransferase genes *Dnmt1*(**B**), *Dnmt3a* (**C**), and *Dnmt3b* (**D**); (**E**–**G**) expression levels of the methylcytosine dioxygenase genes *Tet1* (**E**), *Tet2* (**E**), and *Tet3* (**G**). (**A**–**G**) Cerebral cortex samples. Values are mean ± SEM of male and female mice ((**A**) *n* = 4–6; (**B**) *n* = 5–8; (**C**) *n* = 6–11; (**D**) *n* = 9–12; (**E**) *n* = 9–11; (**F**) *n* = 10–12; (**G**) *n* = 7–10). Statistics: ANOVA, interaction genotype per treatment effect (G x Tr) in (**A**), effect of genotype (G) in (**E**,**F**), *p* values of the significant factor and factor interaction are indicated in the corresponding graphs; Bonferroni post-hoc test after a significant factor interaction, * *p* < 0.05, ** *p* < 0.01, *** *p* < 0.001 between groups as indicated.

Male and female 5XFAD mice from control litters showed a tendency for higher exploratory activity than WT mice in the open field test (OFT). Horizontal activity is shown in Figure 2A,B. There was a borderline effect of the genotype factor in the total distance ambulated (*p* = 0.0603) and in the mean speed without rest intervals (*p* = 0.0530). However, 5XFAD offspring from TPPU-treated dams showed similar activity to WT mice, although the treatment factor did not reach significance. Therefore, these results suggested the presence of mild hyperactivity in young 5XFAD mice that was reduced by maternal TPPU treatment (Figure 2A,B). Vertical activity was significantly lowered by TPPU treatment in both WT and 5XFAD animals, as shown in Figure 2C. As a whole, a decrease in horizontal and vertical activities may indicate TPPU-induced changes in the exploratory pattern leading to lower excitability in a stressful environment. Finally, no changes were detected in grooming activity which discards the presence of stereotypic-like disorders, as shown in Figure 3D.

### 2.2. Maternal TPPU Treatment Induced Neuroprotective Epigenetic Changes in 5XFAD and WT Offspring

#### 2.2.1. Changes in the Global DNA Methylation Landscape

Epigenetic methylation of DNA silences gene expression by direct methylation of the cytosine carbon ring in the dinucleotide CpG repeats [29]. Impaired global DNA methylation has been associated with AD neuropathology and cognitive loss [30].

The global DNA methylation profile was calculated as the ratio between the 5-methylcytosine (5-mC) and 5-hydroxymethylcytosine (5-hmC) levels and the results are displayed in Figure 3A. There was an interaction between the ANOVA factors genotype and treatment, indicating that maternal TPPU differently affected WT and 5XFAD mice. That is, it increased DNA methylation in WT mice but normalized the excessive methylation shown by 5XFAD mice. The gene expression of the specific DNA methyl transferase (DNMT) enzymes *Dnmt1*, *Dnmt3a,* and *Dnmt3b* is shown in Figure 3B–D, respectively, with no significant changes in any of them. However, ten-eleven translocation family (TET) genes *Tet1* and *Tet2* showed an effect of genotype indicating higher levels in 5XFAD mice. The statistical results and several trends shown in the displayed graphs indicate that there were global 5-mC differences between 5XFAD and WT offspring and the maternal treatment with TPPU would mitigate the 5XFAD epigenetic alterations.

#### 2.2.2. Changes in the Histone Deacetylation Machinery

**Figure 4:**
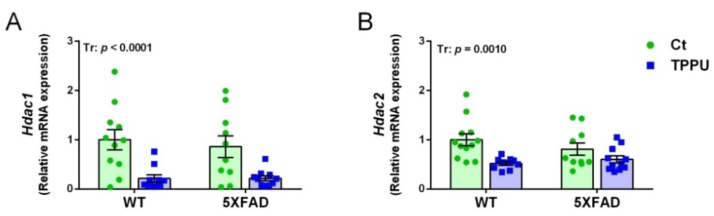
Histone deacetylation changes in the brain tissue of 2-month-old WT and 5XFAD mice from litters with maternal TPPU treatment or control (Ct). Expression levels of the histone deacetylase genes *Hdac1* (**A**) and *Hdac2* (**B**). (**A**,**B**) Hippocampus samples. Values are mean ± SEM of male and female mice ((**A**) *n* = 10–11; (**B**) *n* = 10–12. Statistics: ANOVA, effect of treatment (Tr) in (**A**,**B**), *p* values of the significant factor are indicated in the corresponding graphs.

Several histone deacetylase enzymes (HDACs) have been implicated in memory and cognition [31]. Therefore, we analyzed the brain expression of the genes *Hdac1* and *Hdac2* by qPCR. The mRNA of these epigenetic enzymes was significantly decreased by maternal TPPU treatment in both 5XFAD and WT offspring, as shown in Figure 4A,B. However, no genotype differential effect was detected in the young male and female mice. Removal of acetyl groups from lysine residues on histones increases chromatin compaction into a transcriptionally repressed state. Therefore, TPPU-induced downregulation of *Hdac1* and *Hdac2* would activate gene transcription.

### 2.3. Maternal TPPU Treatment Did Not Modify Amyloid β_42_ but It Protected against Tau Hyperphosphorylation in 5XFAD Offspring

**Figure 5:**
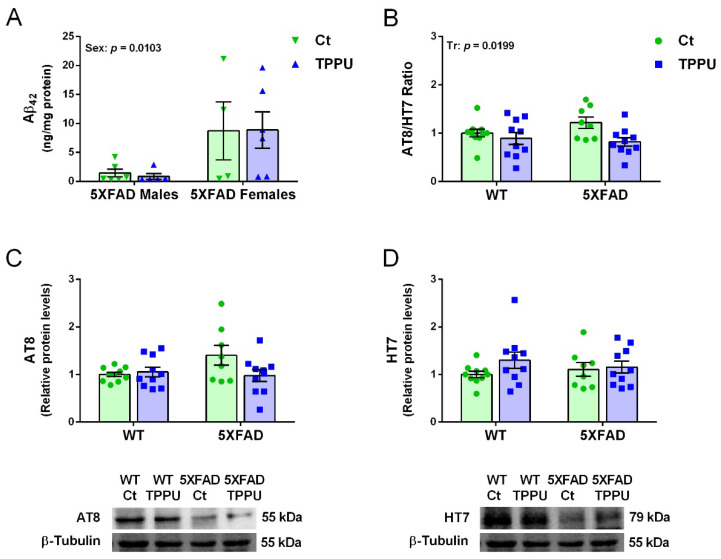
Amyloid β_42_ (Aβ_42_) and hyperphosphorylated tau (p-tau) in 2-month-old 5XFAD mice from litters with maternal TPPU treatment or control (Ct). (**A**) Differential Aβ_42_ concentration in male and female 5XFAD mice; (**B**) ratio of protein levels of p-tau immunodetected by AT8 antibody (values shown in (**C**)) and total tau immunodetected by HT7 antibody (values shown in (**D**)) in WT and 5XFAD offspring. Representative blots are shown below the corresponding histograms in (**C**,**D**). (**A**) Cerebral cortex samples; (**B**–**D**) hippocampus samples. Values are mean ± SEM ((**A**) *n* = 4–6; (**B**–**D**) *n* = 8–10). Statistics: ANOVA, effect of sex (Sex) in (**A**) and treatment (Tr) in (**B**), *p* values of significant factors are indicated in the corresponding graphs; borderline interaction effect (G × Tr) in (**C**) (see text).

Female 5XFAD mice showed higher levels of amyloid β_42_ quantified by ELISA in brain homogenates than males as shown in Figure 5A. These differential levels at 2 months of age agree with those reported in the characterization of the strain (5XFDA, line Tg6799 [32]. The high dispersion of results at this early age might mask any treatment effect. However, TPPU treatment induced a general decrease in hyperphosphorylated tau (p-tau) as shown by the significant effect of treatment in the p-tau/total tau ratio (Figure 5B). Male and female 5XFAD mice showed similar levels of p-tau and the data were analyzed together. Levels of p-tau were low as expected at this age in a strain with predominantly amyloid pathology. However, there was a trend to higher p-tau in 5XFAD mice that was suppressed by maternal TPPU treatment as shown in Figure 5B,C. Furthermore, the interaction between genotype and treatment reached a borderline significance in p-tau protein levels when normalized by the loading control (*p* = 0.0638, Figure 5C). Likewise, total tau levels were similar in the different experimental groups (Figure 5D). Thus, there was incipient p-tau pathology protected by maternal TPPU.

### 2.4. Maternal TPPU Treatment Decreased Neuroinflammation in the Offspring Mice

**Figure 6:**
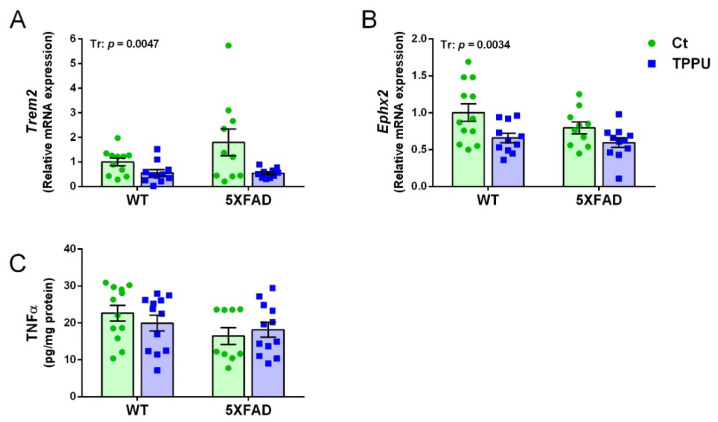
Neuroinflammatory markers in 2-month-old 5XFAD mice from litters with maternal TPPU treatment or control (Ct). Expression levels of the genes *Trem2* (**A**) and *Ephx2* (**B**); (**C**) tumor necrosis factor α (TNFα) concentration (pg/mL). (**A**,**B**) Hippocampus samples; (**C**) cerebral cortex samples. Values are mean ± SEM ((**A**) *n* = 10–11; (**B**) *n* = 10–12; (**C**) *n* = 9–12). Statistics: ANOVA, effect of treatment (Tr) in (**A**,**B**), *p* values of the significant factor are indicated in the corresponding graphs.

A relevant microglial gene activated in AD is the triggering receptor expressed on myeloid cells 2 (*Trem2*). Expression of *Trem2* showed a trend to increase in 2-month-old 5XFAD mice as shown in Figure 6A. High dispersion of the data probably caused a lack of genotype significance. Interestingly, offspring of TPPU-treated dams showed a significant decrease of *Trem2* mRNA levels. Seven-month-old 5XFAD showed an 8.8-fold increase in *Trem 2* compared to age-matched WT levels (Supplementary Figure S1A). Regarding the gene epoxide hydrolase 2 (*Ephx2*) that codifies for the sEH enzyme, there is a decrease in the TPPU litters as shown in Figure 6B. This unexpected effect may contribute to decreased neuroinflammation in the offspring brain. Older 5XFAD mice did not show changes in the *Ephx2* gene (Supplementary Figure S1B). Gene expression levels of the cytokine interleukin 6 (*Il6*) and the chemokine C-C motif ligand 3 (*Ccl3*) were similarly low in all of the young mice (not shown). However, analysis of these markers in -the added reference group of 7-month-old 5XFAD mice showed respective fold increases of 2.3 and 36.6 compared to WT mice (Supplementary Figure S1C,D). Finally, the protein concentration of tumor necrosis factor α (TNFα) was similar in the different experimental groups. Therefore, despite a low brain inflammatory status at this early age, there was a protective effect of maternal TPPU treatment. Interestingly, the decrease in neuroinflammatory markers in the offspring was a specific TPPU effect, as it was not mediated by the lower inflammatory status of the dams (plasma levels of C reactive protein (CRP) and TNFα of female progenitors are shown in Supplementary Table S1).

### 2.5. Female Mice Showed Higher Gliosis and Neurodegenerative Changes Than Male Mice

**Figure 7:**
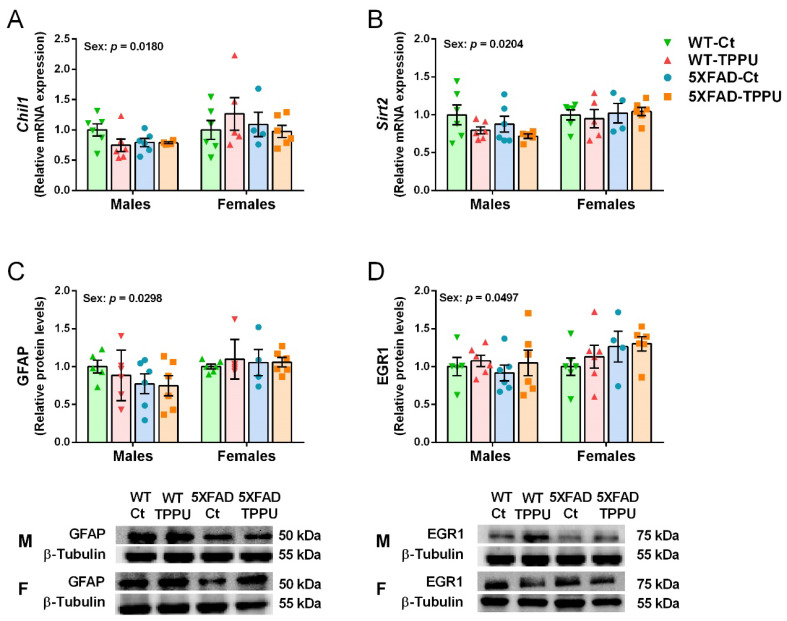
Differential neurodegenerative markers in male and female 5XFAD mice. Expression levels of the genes *Chil1* (**A**) and *Sirt2* (**B**). Relative protein levels of glial fibrillary acidic protein (GFAP) (**C**) and early growth response 1 protein (EGR1) (**D**). Representative blots for males (M) and females (F) are shown below the corresponding histograms in (**C**,**D**). (**A**,**B**) Hippocampus samples; (**C**,**D**) cerebral cortex samples. Values are mean ± SEM ((**A**–**D**) *n* = 4–6). Statistics: ANOVA, effect of sex in (**A**–**D**), *p* values of the significant factor are indicated in the corresponding graphs. The Bonferroni post-hoc test was not significant in the comparison between specific experimental groups of males and females.

Gene expression of the neurodegenerative markers chitinase-like 1 (*Chil1)* and sirtuin 2 (*Sirt2*) showed a significant effect of sex and the experimental groups of males and females were analyzed separately. No effect of genotype or treatment was detected for these genes as shown in Figure 7A,B. However, older male 5XFAD mice showed a 1.8-fold increase in *Chil1* mRNA and a 1.2-fold increase in *Sirt2* mRNA (Supplementary Figure S1E,F). Protein levels of the reactive astrocyte marker glial fibrillary acidic protein (GFAP) also showed a differential effect of sex and no further statistical differences as indicated in Figure 7C. Finally, protein levels of the neuroplasticity transcription factor early growth response 1 protein (EGR1) were also differential in male and female mice as shown in Figure 7D. Generally, females showed higher levels of these markers than males, suggesting a greater susceptibility to neurodegenerative processes. Interestingly, female responses were not linked to specific treatment or genotype as shown by the absence of statistical differences in the post-hoc analysis of the four markers (Figure 7A–D).

## 3. Discussion

The present study examined the effects of a pharmacological maternal intervention with TPPU during pregnancy and lactation, showing that it could prevent or delay the course of AD in offspring that inherited familial AD mutations. Behavioral performance and underlying changes in epigenetic mechanisms and AD hallmarks were analyzed. Total preservation of learning and memory induced by maternal TPPU in the 2-month-old offspring with the 5XFAD genotype is remarkable. Cognitive loss in these mice is induced by early amyloidosis from 1.5–2 months of age linked to five familial AD mutations, as reported in the strain characterization [32]. These authors did not detect impairments in the Y-maze at that early age. However, our current results of early deficits of recognition memory in the ORT and spatial memory in the OLT agree with previous findings of 5XFAD deficits in the Morris water maze test at 2 months of age [33]. Furthermore, the decrease of a hyperactivity trend by TPPU indicates maintenance of brain circuitry. Hyperactivity later turns to decreased motor activity in AD mice as seen in the reported comparison between 2-month-old and 8-month-old 5XFAD [33]. This mild neuropathology sign may be related to synaptic excitability reported in other mouse models [34] and the neuropsychiatric symptom of agitation, common in AD patients [35]. Here the treatment with TPPU, as an inhibitor of the sEH enzyme, would increase the maternal endogenous levels of EET arachidonic acid metabolites to exert its beneficial effects. In addition, TPPU is expected to reach fetuses and lactating pups as it crosses the blood–brain barrier [36]. As mentioned above, there is no doubt about the importance of maternal influences on offspring neurodevelopment [37]. For instance, dietary supplementation of rat dams with the essential PUFAs linoleic and linolenic fatty acid during gestation and lactation was reported to anticipate reflex maturation and improve memory in the offspring [38]. However, reports of beneficial effects of pharmacological maternal treatments on offspring at risk for brain disease are scarce. In a similar experimental setting, TPPU has protected juvenile mice from neuropsychiatric-like traits induced by maternal infection during pregnancy [39].

In the epigenetic analysis, our finding of higher global DNA methylation as the 5-mC/5-hmC ratio in the 2-month-old 5XFAD mice compared to the WT mice suggests an abnormal increase in DNA methylation leading to a decrease in gene transcription. In addition, relative gene expression of both DNMTs and TETs enzymes compared to WT mice generally showed a tendency to increase as compared to WT. These results are in agreement with previous findings in 5XFAD at this young age [33]. Furthermore, accumulation of both 5-mC and 5-hmC with increasing age has been reported in an AD mouse model [40]. Interestingly, maternal TPPU reduced the 5XFAD 5-mC/5-hmC ratio to WT levels and on the contrary increased that of WT. In the end, offspring of both genotypes of TPPU-treated dams reached similar levels. DNA methylation is a major regulator of gene transcription [41] and an aberrant DNA methylation pattern is known in AD brain [42]. It is known that an improved regulation of this epigenetic mechanism during development benefits synaptic plasticity and cognitive function in the offspring [41]. This may be the case here, as we found an effect of the treatment factor in memory tests, corroborating the general cognitive enhancement in the offspring of TPPU-treated dams.

On the other hand, we did not detect changes in the expression of the genes *Hdac1* and *Hdac2* in 5XFAD young mice compared to WT mice. However, some authors have reported HDAC2 elevation in mouse models and AD brain and demonstrated its involvement in the blockade of cognitive functions [33,43]. Other authors reported a decrease in HDAC1 and 2 in AD brain [44] that may be associated with advanced pathology. Interestingly, general inhibition of *Hdac1* and *Hdac2* by maternal TPPU may induce preventive and protective mechanisms against neurodegeneration in WT and 5XFAD mice, respectively. Histone deacetylation is a crucial epigenetic mechanism that regulates gene expression and can modulate neurodevelopment and brain function [45]. Particularly, dysregulation of the transcriptional repressors HDAC1 and 2 are important drivers of neurodegeneration and AD [42,46]. HDAC1 and 2 are negative regulators of synaptic plasticity and memory formation and circumventing its epigenetic blockade would be beneficial against neurodegeneration [47,48]. Inhibition of both HDAC1 and 2 has shown synaptic improvement in experimental systems that model the early stages of AD [49]. In addition, HDAC inhibition has been shown to be protective against Aβ-induced hyperphosphorylation of tau through inhibition of tau kinase enzymes [50].

The epigenetic changes induced by maternal TPPU in 5XFAD offspring suggest involvement of epigenetic signaling in the modulation of AD traits during brain development. Furthermore, epigenetic changes in WT offspring suggest improved neurodevelopment as an acquired resilience against neurodegeneration. The implications of epigenetic changes in the initiation and development of AD are not well understood, but they may fill the gap between genetic and environmental risk [42].

Remarkably, we found a decrease in p-tau by maternal TPPU treatment. However, TPPU did not decrease genetically-driven Aβ_42_ in 5XFAD offspring. Tau hyperphosphorylation is induced by proinflammatory mediators released by reactive microglia under amyloid pathology [51]. Therefore, we hypothesize that both HDAC inhibition of tau kinase activity and the absence of microglial pro-inflammatory mediators, as discussed below, decreased p-tau levels downstream of Aβ generation in the 2-month-old mice. Cognitive benefices paralleling p-tau decrease in the offspring are in agreement with the fact that PET detection of tau tangles is the pathological sign most consistent with cognitive impairment and neurodegeneration in AD patients [52].

Neuroinflammation was detected at the age of 2 months as a trend towards an increase in the microglial activation marker gene *Trem2*. However, neuroinflammation was a prominent sign in the 7-month-old 5XFAD mice, in agreement with its relevance in AD triggering and progression [53,54]. Therefore, polarization of microglia to a reactive phenotype is an early event after Aβ_42_ accumulation in these mice. *TREM2* has also been found increased in the cerebral cortex of autosomal dominant AD patients [55]. Microglia play an important role in the innate immune response of the brain and their phenotypic changes may protect against neurodegeneration or worsen it when chronically activated [55,56]. Interestingly, maternal TPPU maintained low levels of *Trem2* in both 5XFAD and WT mice. Microglia plasticity is modulated by epigenetic modifications including DNA methylation and histone acetylation reported here [57,58]. Therefore, we may speculate that microglia were imprinted early by epigenetic changes induced by TPPU treatment to a neuroprotective phenotype, although further investigations would be required to confirm the involvement of either HDACs inhibition or specific DNA gene methylation changes.

Strikingly, TPPU treatment during brain development induced long-term downregulation of the sEH gene *Ephx2*. This would maintain an anti-inflammatory milieu in the brain of the offspring. Several epigenetic mechanisms may modulate *Ephx2* expression, including DNA methylation [59]. On the other hand, no change in the peripheral inflammatory marker CRP [60] was found in the dams at the end of their treatment with TPPU.

Finally, the increase in female mice in four biomarkers of neurodegeneration compared to males indicates early sex differences in the mouse phenotype. Expression of the genes *Chil1* and *Sirt2* was increased in the 7-month-old 5XFAD mice as compared to the WT mice, but we found a general increase in young female offspring as compared to the males. Sirtuin 2 is a histone deacetylase belonging to the sirtuin family. In contrast to other sirtuins, sirtuin 2 promotes neurodegeneration in experimental models [61] and some *SIRT2* variants increase AD risk [62]. Chitinase-like 1 is a marker of reactive gliosis and the innate immune response which shows human counterpart (chitinase 3-like 1 or YKL-40) increases in the CSF of AD patients, although changes in postmortem AD brain are controversial [63,64]. Young female offspring also showed a general increase in protein levels of GFAP and EGR1 as compared to the male offspring. The reactive astrocyte marker GFAP is increased in AD brain and in AD mouse models [32,63]. GFAP and chitinase-like 1 are differentially related to AD but both may mediate brain atrophy and cognitive impairment [65]. In addition, indicating some neurodegeneration risk, the female increase in EGR1 may be analogous to the reported increase in EGR1 transcript preceding pathology in the early stages of AD brain [66]. Next, there is a decreased expression of this neuroplasticity gene with advanced pathology as we previously reported in 7-month-old 5XFAD mice [17]. A mild increase in these AD-related pathological markers in 2-month-old female mice compared to male mice is consistent with a higher risk of developing AD in the female sex. Indeed, women are more likely to suffer AD than men [67,68].

## 4. Materials and Methods

### 4.1. Animal Breeding and Experimental Design

Heterozygous 5XFAD male mice were bought from Jackson Laboratories (Bar Harbor, ME, USA) and B6SJLF1/J hybrid female mice were bought from JANVIER LABS (Le Genest-Saint-Isle, France), Male 5XFAD and female B6SJLF1/J were crossed to breed WT and 5XFAD offspring, following the breeding directories from Jackson Laboratories. Each male was mated overnight with two females. Then, each female was transferred to a new cage to start the treatment.

TPPU (Sigma-Aldrich, Darmstadt, Germany) was administered orally within the drinking water during pregnancy and lactation, mixed with cyclodextrin 3% (Sigma-Aldrich) to improve its solubility. Control females had the same dose of cyclodextrin in the drinking water. TPPU dose was decided according to previous studies from our group 1; hence, treated females were ensured with a constant daily dose of 5 mg/kg of TPPU. For this purpose, the concentration of the compound in the drinking water was adjusted twice per week according to the body weight and water consumption. Six TPPU-treated and six control dams and their respective litters remained undisturbed until pup weaning at 21 days of age. All pups were sexed and identified by ear punching. Tissue samples obtained from ear punches were used for genotype identification by PCR. Mice were housed according to sex and litter. No further treatment was administered to the offspring.

**Figure 8:**
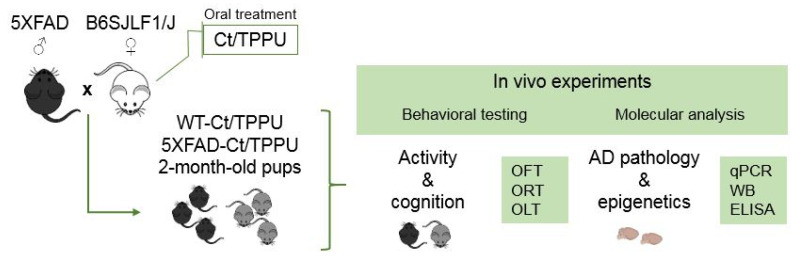
Experimental design scheme. Two-month-old WT and 5XFAD offspring of dams treated with vehicle (Ct) or TPPU were submitted to behavioral testing and molecular analysis. Abbreviations: ELISA: enzyme-linked immuno sorbent assay, OFT: open field test, OLT: object location test, ORT: object recognition test, qPCR: real-time quantitative polymerase chain reaction, WB: Western blotting.

Male and female offspring at 2 months of age were used in this study. The total of 71 animals obtained from the control or TPPU-treated dams were distributed into the corresponding experimental groups as follows: WT Control (WT-Ct), *n* = 7 males and *n* = 7 females; WT TPPU (WT-TPPU), *n* = 10 males and *n* = 10 females; 5XFAD Control (5XFAD-Ct), *n* = 7 males and *n* = 5 females; 5XFAD TPPU (5XFAD-TPPU), *n* = 13 males and *n* = 12 females. All of the mice were submitted to behavioral testing and then euthanized to obtain brain tissue for molecular analysis. A scheme of the experimental design is shown in Figure 8.

A group of untreated male 5XFAD mice (*n* = 8) at the age of 7 months with advanced AD pathology and an age-matched group of WT mice (*n* = 8) were added to the study as a reference for selected molecular analysis of brain tissue.

Animal procedures were carried out in the Animal Unit of the University of Barcelona (UB), Spain. The mice were housed in Makrolon^®^ cages (Tecniplast, Buguggiatta, Italy), with free access to food and water and a room temperature maintained at 22 ± 2 °C and a 12 h light/12 h dark cycle. Approval for the study and experimental protocols was confirmed by the local animal experimentation ethics committee (CEEA-UB) under the guidelines of the Animal Experimentation Commission of the Autonomous Government of Catalonia (approval reference: 22/20 from date 5 November 2020). All procedures were performed in accordance with the European Commission Council Directive 86/609/EEC on this subject.

### 4.2. Sample Collection

Immediately after the pups were weaned, the dams were anesthetized with isoflurane to obtain blood samples by cardiac puncture and euthanized. The blood was collected in heparinized tubes and centrifuged for 10 min at 2000× *g* using a refrigerated centrifuge. The plasma was collected and stored at −80 °C until testing.

Brain tissue samples were obtained from 2-month-old mice at necropsy. The brain was dissected out on a cold plate, and the cerebral cortex (CC) and hippocampus (HC) tissue were snap-frozen in liquid nitrogen and stored at −80 °C until molecular testing. Relative expression of specific genes and semiquantitative protein levels were analyzed in both CC and HC samples with generally similar results. For better clarity, the results of one of the regions were selected as indicated in the figure legends. HC tissue from 7-month-old mice was used as a reference for the expression of selected genes. ELISA determinations were performed in CC.

### 4.3. Behavioral Procedures

Open field (OF). Locomotor activity and general behavior were analyzed in a 50 × 50 × 20 cm white maze. Each mouse was gently placed in the center of the arena and allowed to move freely for 5 min. A computerized video tracking system (SMART v3.0, Panlab S.A., Barcelona, Spain) was used to measure the traveled distance and the ambulatory pattern. Rearing and grooming activities were also analyzed.

Object recognition test (ORT). The assessment of recognition memory is based on the mouse′s tendency to explore novel objects longer than familiar ones. Each mouse went through successive 10-min sessions in a 30 × 40 × 30.5 cm black maze. In the habituation, the mouse was allowed to explore the arena without any objects for two consecutive days. The next day, in the acquisition trial, the mouse was allowed to explore two identical plastic objects (A + A) that were located equidistant from the corners. Two h later, the mouse was tested in the retention trial where one of the objects was replaced by another object with a different shape and color (A + B). All of the sessions were videotaped and blindly analyzed to measure the exploration time (t) of each object. Object exploration was defined as the orientation of the nose to the object at a distance smaller than 2 cm. A discrimination index was calculated as [novel object (t)—familiar object (t)]/[total (t) novel + familiar].

Object location test (OLT). The assessment of spatial memory is based on the mouse’s tendency to explore a newly located object for a longer time than an unmoved object. The test uses the same apparatus and general procedure as ORT with some modifications. The habituation and test sessions lasted 5 min. In the acquisition trial, the mouse explored two identical objects (A1 + A2) located equidistant from each other and near one of the long walls. In the 2 h retention trial, the mouse was allowed to explore the same objects but one of them was moved to a position near the opposite wall (A1 + A3). In OLT, the DI was calculated as [novel location (t)—familiar location (t)]/[total (t) novel + familiar].

### 4.4. Real-Time Quantitative Polymerase Chain Reaction (qPCR)

Extraction of total RNA from the brain tissue samples was carried out using a mirVanaTM mRNA Isolation Kit (Applied Biosystems, Foster City, CA, USA) following the manufacturer′s protocol. RNA content was determined using a ND-1000 spectrophotometer (NanoDrop Technologies, Wilmington, DE, USA).

Reverse transcription from RNA to first-strand complementary DNA (cDNA) was performed using a High-Capacity cDNA Reverse Transcription Kit (Thermo Fischer Scientific, Waltham, MA, USA). Three hundred ng of RNA per sample were loaded in a thermal cycler (FlexCycler, Analytikjena, Jena, Germany). cDNA samples were stored at −20 °C until use.

Gene expression was analyzed by qPCR using TaqMan^®^ Fluorescein amidite (FAM)-labeled specific probes (Thermo Fisher Scientific, Waltham, MA, USA) and a Quantimix Easy Probe kit (Biotools, Madrid, Spain). The reaction mix containing 6.75 ng of cDNA was loaded in a CFX96TM Real-Time System (Bio-Rad, Hercules, CA, USA). Alternatively, SYBR Green qPCR was performed in a Step One Plus Detection System (Applied Biosystems). Each reaction mixture contained 6.75 μL of cDNA (which concentration was 2 μg), 0.75 μL of each primer (the concentration of which was 100 nM), and 6.75 μL of SYBR Green PCR Master Mix (2X) (Applied Biosystems). The Taqman assay probes and primers used are listed in Supplementary Tables S2 and S3, respectively. The samples were analyzed in duplicate.

The results were normalized to actin beta (*Actb*) gene expression using the comparative cycle threshold (2^−ΔΔCT^) method.

### 4.5. Western Blotting (WB)

Protein extracts were obtained from the brain tissue samples. The tissues were homogenized in a cold RIPA lysis buffer supplemented with protease and phosphatase inhibitors, ultrasonicated, and centrifuged. The protein concentration of the supernatants was determined using the Bradford protein assay (Bio-Rad). Forty μg of protein per sample were denatured by boiling, loaded into polyacrylamide gels, and separated by SDS-PAGE at 100 V. Electrophoresed proteins were transferred to PVDF membranes by electroblotting at 200 mA for 90 min. Then, the membranes were incubated with a blocking buffer for 1 h followed by 4 °C overnight incubation with primary antibodies. Secondary antibodies were peroxidase-conjugated. The antibodies used are listed in Supplementary Table S4. The proteins were visualized by enhanced chemiluminescence detection in a Chemidoc™ Imaging System (Bio-Rad). The densitometric analysis was performed using Image Lab software (v3.0.1, Bio-Rad). The protein levels were normalized using actin, β-tubulin, or glyceraldehyde 3-phosphate dehydrogenase (GAPDH).

### 4.6. Enzyme-Linked Immunosorbent Assay (ELISA)

The plasma levels of CRP were measured with the Mouse CRP ELISA Kit (Invitrogen, Thermo Fisher Scientific).

TNFα was measured with the Mouse TNF alpha uncoated ELISA kit (Invitrogen, Thermo Fisher Scientific) in plasma and brain tissue samples.

Global DNA methylation was determined with the MethylFlash Methylated DNA 5-mC Quantification kit (Colorimetric) (EpiGentek, Farmingdale, New York, NY, USA). Global DNA hydroxymethylation was determined with the MethylFlash Global Hydroxymethylation (5-hmC) ELISA Easy Kit (Colorimetric) (EpiGentek). DNA methylation and hydroxymethylation were measured in brain samples.

The Human Aβ_42_ ELISA Kit (Invitrogen, Thermo Fisher Scientific) was used to quantify Aβ_42_ in 5XFAD mouse brains.

Sandwich ELISA assays were performed according to the specific manufacturer′s instructions. Absorbances were measured in a spectrophotometer Multiskan SkyHigh (Thermo Fisher Scientific) and the corresponding concentrations were calculated from standard curves. The samples were tested in duplicate.

### 4.7. Statistical Analysis

The results are expressed as mean ± SEM. The normality of the data was tested with the Shapiro–Wilk test. The data were analyzed by ANOVA with the factors: sex, genotype, and treatment. In the absence of a sex effect, male and female data were jointly analyzed by two-way ANOVA (main factors: genotype and treatment, and its interaction). The presence of a statistical significance of the ANOVA factors or its interaction is indicated in the figures. In this two x two ANOVA design, a comparison between the group means was performed after a significant interaction of main factors using Bonferroni′s post-hoc test. In the presence of a sex effect, male and female experimental groups were analyzed separately. We first checked for genotype or treatment effects for each sex. In their absence, we analyzed the male and female data by two-way ANOVA (main factors: sex and experimental group, and its interaction). The Bonferroni post-hoc test was used to discern whether sex differences could be attributed to any of the four experimental groups. The Student′s t-test was used to analyze the significance between the two experimental groups, where indicated (supplementary groups of 7-month-old animals). Statistical outliers were identified by Grubbs′ test (α = 0.5). The results were considered significant when the *p*-value < 0.05. The data were analyzed using the GraphPad Prism 6.01 package (GraphPad Software, Inc, La Jolla, CA) and IBM SPSS Statistics v23 (IBM Corp., Armonk, New York, NY, USA).

## 5. Conclusions

Two-month-old 5XFAD mice showed memory loss and mild AD-like neuropathology comparable to the early stages of AD. A maternal pharmacological intervention with TPPU, which increases endogenous EETs, protected the mice against cognitive impairment and reduced p-tau and microglial reactivity. This protection was achieved despite sustained Aβ_42_ generation driven by familial AD mutations in the 5XFAD mouse model. Changes in 5-mC/5-hmC and histone deacetylase machinery in both WT and 5XFAD offspring demonstrated that the epigenetic mechanism substantially contributed to brain resilience and neuroprotection, thus reinforcing the importance of beneficial maternal interventions. Furthermore, female offspring mice reproduced the higher AD risk described in women, as shown by higher amyloid levels in 5XFAD mice and increased AD risk markers in all of the females. Hence, the results obtained may contribute to understanding the connection between early events and late neurodegeneration and designing effective therapies to increase brain resilience and prevent AD. Finally, the interest of a time-course study in the offspring should be considered to confirm the maternal TPPU neuroprotection found in early AD up to advanced pathological stages.

## Data Availability

All data are included in the article and Supplementary Materials.

## Supplementary Materials

The following supporting information can be downloaded at: https://www.mdpi.com/article/10.3390/ijms232315151/s1.
