# Supplementary material for: Neuroprotective Epigenetic Changes Induced by Maternal Treatment with an Inhibitor of Soluble Epoxide Hydrolase Prevents Early Alzheimer′s Disease Neurodegeneration
Source: Int J Mol Sci. 2022 Dec 2;23(23):15151. doi: 10.3390/ijms232315151 (PMC9740580; doi:10.3390/ijms232315151)
Supplement: Supplementary file 1 [file ijms-23-15151-s001.zip › ijms-2029859-supplementary.pdf]

International Journal of *Molecular Sciences*

MDPI

Supplementary Materials

Neuroprotective epigenetic changes induced by maternal treatment with an inhibitor of soluble epoxide hydrolase prevents early Alzheimer's disease neurodegeneration

Clara Bartra, Alba Irisarri, Ainhoa Villoslada, Rubén Corpas, Samuel Aguirre, Elisa García-Lara, Cristina Suñol, Mercè Pallàs, Christian Griñán-Ferré, Coral Sanfeliu.

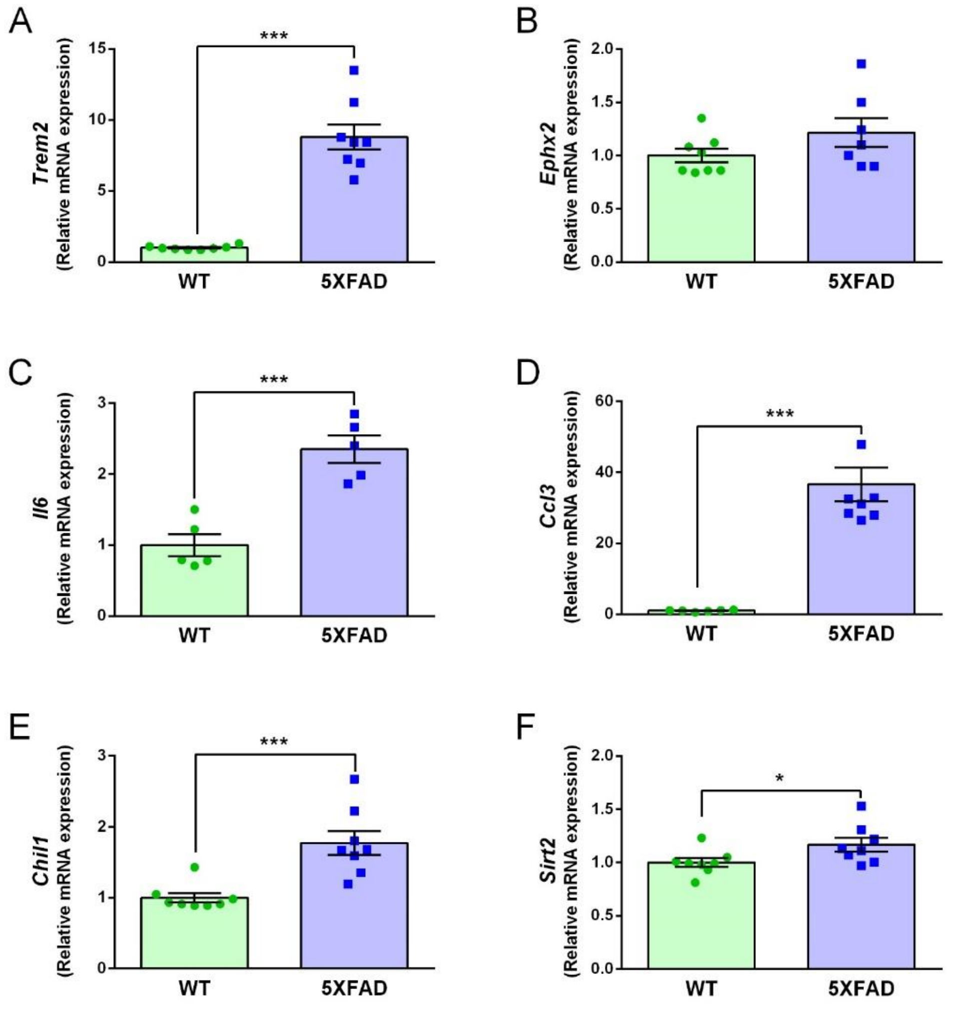

Figure S1. Gene expression of inflammatory and neurodegenerative biomarkers in the hippocampus of 7-month-old 5XFAD male mice. Increased relative mRNA values of 5XFAD mice compared to wild-type (WT) littermates of the genes: (**A**) Triggering receptor expressed on myeloid cells 2, *Trem2*; (**B**) Epoxide hydrolase 2, *Ephx2*; (**C**) Chitinase-like 1, *Chil1*; (**D**) Sirtuin 2, *Sirt2*; (**E**) Interleukin 6, *Il6*; (**F**) Chemokine C-C motif ligand 3, *Ccl3*. Values are mean ± SEM (n = 5 - 8). Statistics: Student's t test, *$p < 0.05$, ***$p < 0.001$.

International Journal of *Molecular Sciences*

MDPI

Table S1. CRP and TNFα ELISA results from mouse dams plasma samples

		Ct	TPPU
**CRP**	μg/mL (mean ± SEM)	10.36 ± 0.81	12.77 ± 1.36
	n	6	6
**TNFα**	pg/mL (mean ± SEM)	< 2	< 2
	n	6	5

Abbreviations: CRP, C-reactive protein; TNFα, Tumor necrosis factor α.

Table S2. TaqMan Assays used in qPCR analysis

Gene Name	Gene Symbol	Assay ID
Actin, beta	*Actb*	Mm02619580_g1
Brain derived neurotrophic factor	*Bdnf*	Mm04230607_s1
Chemokine C-C motif ligand 3	*Ccl3*	Mm00441259_g1
Chitinase-like 1	*Chil1*	Mm00801477_m1
Epoxide hydrolase 2	*Ephx2*	Mm01313813_m1
Histone deacetylase 1	*Hdac1*	Mm02391771_g1
Histone deacetylase 2	*Hdac2*	Mm00515108_m1
Interleukin 6	*Il6*	Mm00446191_m1
Sirtuin 2	*Sirt2*	Mm01149204_m1
Triggering receptor expressed on myeloid cells 2	*Trem2*	Mm00451744_m1

Table S3. Primers used in SYBR Green qPCR analysis

Gene name	Gene symbol	Forward primer (5′-3′)	Reverse primer (5′-3′)
Actin, beta	*Actb*	CAACGAGCGGTTCCGAT	GCCACAGGTTCCATACCCA
DNA methyltransferase (cytosine-5) 1	*Dnmt1*	GGGCTGTGCTTCCTGTCG	GGTGTCCCCAAGCTTGTTCT
DNA methyltransferase 3A	*Dnmt3a*	GGGCCACACGGCAGAG	CACGGTTCTCCTCCTGTTCC
DNA methyltransferase 3B	*Dnmt3b*	TGCCAGACCTTGGAAACCTC	GCTGGCACCCTCTTCTTCAT
Tet methylcytosine dioxygenase 1	*Tet1*	CCTGCCTCTTCTACGGGAAC	GATTTGGAAGGCTTTGCGGG
Tet methylcytosine dioxygenase 2	*Tet2*	CCATCATGTTGTGGGACGGA	ATTCTGAGAACAGCGACGGT
Tet methylcytosine dioxygenase 3	*Tet3*	GGGCAGGCAGCGTAGC	CAGGATCTGGGGCAAGACAG

Table S4. Antibodies used for Western blot analysis

Antibody	Host	Source (catalog)	WB dilution
Actin	Rabbit	Sigma-Aldrich (#A5060)	1:10,000
Early growth response protein 1 (EGR-1)	Rabbit	Cell Signaling (#4153)	1:1000
Glyceraldehyde 3-phosphate dehydrogenase (GAPDH)	Mouse	Assay designs (#CSA-335)	1:5000
Glial fibrillary acidic protein (GFAP)	Mouse	Sigma-Aldrich (#G3893)	1:500
p-Tau clone AT8	Mouse	Thermo Fisher Scientific (#MN1020)	1:1000
ß-Tubulin	Mouse	Abcam (#ab21754)	1:10,000
Total tau clone HT7	Mouse	Thermo Fisher Scientific (#MN1000)	1:1000
Sheep-anti-mouse HRP conjugated		Amersham (GE) (#NA931)	1:2000
Donkey-anti-rabbit HRP conjugated		Amersham (GE) (#NA934)	1:2000
